# Learning Mechanisms in Alcohol Use Disorders

**DOI:** 10.1111/adb.70168

**Published:** 2026-06-16

**Authors:** Andreas Heinz, Selin Icoez, Yvonne Treusch, Gaetano di Chiara, Melissa G. Halil

**Affiliations:** ^1^ Department of Psychiatry and Psychotherapy, Tübingen Center for Mental Health (TüCMH), Medical School and University Hospital Eberhard Karls University of Tübingen Tübingen Germany; ^2^ German Center for Mental Health (DZPG) Tübingen Germany; ^3^ Department of Psychosomatic Medicine Charité Universitätsmedizin Berlin, Charité Campus Mitte (CCM) Berlin Germany; ^4^ German Center for Mental Health (DZPG) Berlin Germany; ^5^ Department of Health and Social Affairs HSD University of Applied Sciences Cologne Germany; ^6^ Department of Biomedical Sciences University of Cagliari, Cittadella Universitaria di Monserrato Cagliari Italy

**Keywords:** alcohol use disorders, behavioural interventions in addiction, dopamine dysregulation, neuroadaptation in addiction, reward learning mechanisms

## Abstract

Pavlovian and operant learning mechanisms promote and maintain alcohol intake despite aversive consequences. Here, we review the evidence for drug‐associated modifications of basic learning mechanisms during the initiation and maintenance of alcohol use disorders in humans. We discuss how neuroadaptive effects elicited by chronic alcohol intake in the so‐called brain reward system can bias learning and decision making towards drug consumption and contribute to the relapse risk after detoxification. Robustness of results and limitations of the respective neurobiological or imaging techniques are addressed. We opine that studying the effects of alcohol intake on Pavlovian and reinforcement learning can inform psychotherapeutic interventions and help to better understand some new neurobiological interventions. We argue that a focus on drug effects biassing basic learning mechanisms can help to reduce the stigma associated with drug consumption and emphasize behavioural and neurobiological interventions that take individual learning mechanisms into account when promoting alternative coping strategies.

## Graphical abstract

Learning mechanisms mediate the initiation, maintenance, and treatment of AUD.

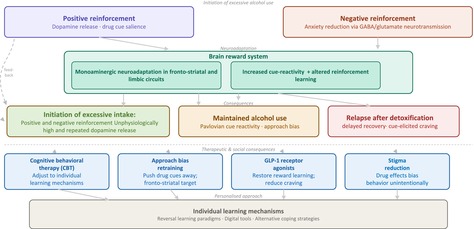

## 1 Introduction

Learning mechanisms and their neurobiological correlates have long been studied in alcohol use disorders (AUDs) [1, 2, 3, 4, 5, 6, 7]. Alcohol consumption can directly reinforce further intake by stimulating endorphin and dopamine release, interacting systems implicated in learning from reward [2, 8] and conditioned cues that drug craving [1, 6]. Alcohol's effects on inhibitory transmitter systems such as GABA may reduce anxiety and contribute to further drug intake via negative reinforcement [9] but have also been observed to increase dopamine release [10, 11].

In a behavioural framework, drug seeking and intake are promoted by unconditioned, rewarding as well as conditioned stimuli, which elicit dopamine release, and the individual thus learns to anticipate and repeat it [1, 10, 11]. Schultz et al. [12, 13] have shown that phasic dopamine release triggered by (initially unexpected) reward is shifted in time towards the conditioned stimulus, predicting that a certain stimulus or action will be rewarded. The neurotransmitter dopamine is assumed to act as a ‘teaching signal’ that attributes incentive motivational value to a conditioned stimulus [1, 6], which, in the case of alcohol addiction, could be a picture of an alcoholic beverage, a bar context or any other drug‐related stimulus [14]. Such stimuli may trigger craving and motivation to drink.

In accordance with the prediction error model of reinforcement learning [12, 13], computational models of reward‐related learning and decision‐making were developed [15, 16] and applied in addiction research [1, 15, 16]. Here, we review alterations in Pavlovian and operant learning mechanisms among persons with AUD. We address habitual and goal‐directed decision making and their respective neurobiological correlates. We discuss the robustness of findings and the limitations of neurobiological and imaging methods in humans. We then compare evidence for theories that try to explain the persistence of drug use despite aversive effects. Finally, we discuss how an understanding of learning mechanisms in drug addiction could contribute to reducing the stigma of drug addiction as well as providing new therapeutic interventions that help subjects learn alternative behaviour strategies.

## 2 Initiation of Excessive Alcohol Use: Positive and Negative Reinforcement

A complex network of social and individual factors contributes to the start and progression of alcohol intake among adolescents in prospective studies [17, 18, 19]. A low level of response to the aversive effects of alcohol intake is a partly heritable trait [17, 18]. Animal experiments and human studies suggested that a low serotonin turnover following social isolation stress and an associated (or genetically predisposed) high availability of midbrain serotonin transporters contribute to a low aversive response to first alcohol experiences [17, 19, 20]. This low aversive response includes low levels of ataxia, sedation and negative mood, potentially mediated by interactions between serotonin and GABAergic neurotransmission in the frontal cortex and by limbic activation to aversive stimuli [19, 21].

Regarding the positive reinforcing effects of alcohol intake, a phasic increase in dopamine release occurs whenever a reward is unexpected or higher than anticipated [12] and attributes salience to stimuli that predict the unexpectedly high reward [6]. Animal experiments showed that drugs of abuse release significantly more dopamine than food intake or sexual encounters [22, 23, 24, 25] in the ventral striatum (including the Nucleus accumbens), a core area of the so‐called brain reward system [22, 26]. Accordingly, there is a ‘supraphysiological’ (i.e., unusually strong) action of drugs on the dopamine neurotransmitter system [14]. This drug effect does not rapidly habituate, as is the case with natural reinforcers [3, 23, 27, 28]. These observations in rodents have been replicated in nonhuman primates with 6 months of alcohol intake, in whom dopamine release was increased in the ventral but decreased in more dorsal parts of the striatum [29].

To test this hypothesis in humans, dopamine release should be measured in vivo. However, human brain imaging techniques do not offer tools to monitor dopamine release in the millisecond range, as observed in animal experiments [12]. One way to indirectly measure dopamine release in vivo is to assess radioligand binding to dopamine receptors, which should be displaced when endogenous levels are increased after drug intake. Acute alcohol intake indeed reduced binding of the radioligand raclopride to dopamine D2 receptors in vivo in the human striatum [30]. This assessment is complicated by differences in radioligand and receptor affinity [31]. The finding was replicated in some but not all human studies and is supported by a recent meta‐analysis [30, 32].

In the context of drug addiction, drug‐associated dopamine release attributes motivationally relevant salience to drug cues [6, 33]. High levels of phasic dopamine release elicited by drugs of abuse and by drug‐associated cues can reinforce further drug seeking and consumption despite aversive consequences [34]. In fact, increased striatal dopamine release was observed among high versus low risk young social drinkers [35]. In young adults, higher dopamine release in the ventral striatum was associated with functional activation of the ventral striatum during reward feedback and with a younger age of first drunkenness [36, 37].

It has long been suggested that the genetic disposition to AUD includes polymorphisms that affect the reinforcing properties of alcohol and other drugs of abuse. Animal models provided evidence that a low availability of dopamine D2 receptors in the Nucleus accumbens may predispose towards impulsive drug intake [38, 39, 40, 41]. In nonhuman primates, dopamine D2 availability decreased with lower social status and was associated with increased drug intake [42, 43, 44, 45]. A lower availability of dopamine D2 receptors may be due to increased competition between radioligands and endogenous dopamine release, as observed in some human studies following stress exposure [46]. On the other hand, first reports in humans were not replicated that genetic variations associated with a low availability of dopamine D2 receptors predispose to the manifestation of AUD [47]. In the prospective ABCD study, alcohol intake in adolescents was not associated with a potentially dopamine‐dependent functional activation of the ventral striatum by reward‐predicting cues [48], not supporting the hypothesis that a deficit in reward processing precedes excessive alcohol use. One study among relatives of persons with AUD instead suggested that a relative increase in dopamine D2 receptors in the ventral striatum and caudate may play a protective role among nonaddicted relatives of persons with alcohol dependence [49]. Altogether, there is currently no convincing evidence that a genetically caused reduction in dopamine D2/3 receptors precedes the development of alcohol addiction in humans.

In patients with Parkinson's disease, ventral striatal dopamine release elicited by reward‐related stimuli was associated with impulsive behaviour [50]. Likewise, alcohol or cue‐induced dopamine release may contribute to impulsive drug intake during the development of AUD [12]. Beyond impulsivity, a series of different factors contribute to AUD development [17, 18]. For example, persons suffering from physical or sexualized violence may consume drugs in a goal‐directed manner to forget for a moment about their experiences. In fact, violent experiences during early development increase both alcohol consumption and mechanisms of dependence more than twofold [51]. Such aversive experiences can also have a sensitizing effect on dopaminergic neurotransmission [52], thus rendering subjects potentially more vulnerable to drug‐related reinforcement.

In summary, strong reinforcement of drug intake and low levels of aversive experiences may help to explain why subjects start to consume excessive amounts of alcohol.

## 3 Maintenance of Excessive Alcohol Use: Pavlovian Mechanisms, Neuroadaptations and Habit Formation

Pavlovian and operant learning mechanisms contribute to the maintenance of alcohol intake in spite of aversive consequences. Drug users often report that drug intake is no longer rewarding but occurs rather as a kind of stimulus‐bound, ‘automatized’ behaviour elicited by drug‐related cues [53]. As one of our patients stated, ‘When I sit at night in front of the TV, with my mailbox full of bills and no partner or sober friend to meet, and I see the beer in front of a lake in a famous alcohol advertisement, I get up and walk to the next gas station where I can buy liquor. When I wake up with a hangover the next day, I feel horrible and experience a strong urge to continue drinking’. Pavlovian learning thus appears to play a role when cues associated with alcohol intake elicit craving and activate brain areas, including the frontal cortex and ventral striatum, which can predict further alcohol intake [54, 55, 56]. Such increased functional activation elicited by alcohol cues did not precede the development of AUD and was not associated with increased impulsivity, suggesting that cue reactivity indeed develops with chronic alcohol intake [57]. Once established, social to heavy beer drinking was associated with increased in vivo dopamine release and functional activation of the right ventral striatum and orbitofrontal cortex upon exposure to beef flavour [58]. Among persons with AUD, alcohol‐associated cues that activate fronto‐striatal networks elicited a habitual approach bias, which can be targeted by systematically training to push drug cues away [59, 60].

A series of animal experiments showed that alcohol seeking and habit formation can be facilitated by neuroadaptive effects and altered neural network functions associated with chronic alcohol intake [34, 61]. In humans, Volkow et al. [62] were among the first to observe that patients with alcohol dependence show a reduced availability of dopamine D2 receptors in the striatum, which may be due to neuroadaptations to excessive drug‐related alcohol release. Several studies were able to replicate this finding [63, 64, 65, 66] and showed that the lower the dopamine D2 receptors, the higher the craving [67, 68, 69, 70] and the functional activation elicited by alcohol cues [71]. Also, several studies that assessed dopamine receptor sensitivity via effects of pharmacological D2 receptor stimulation observed a decrease in dopamine D2 receptor function among persons during early abstinence [72, 73, 74, 75, 76].

Such alterations in dopaminergic neurotransmission can interfere with goal‐directed behaviour [16, 77], which aims at maximizing reward and minimizing aversive experiences. Once a reward is devaluated (e.g., by feeding to satiety), goal‐directed behaviour should stop [78]. Using such devaluation paradigms, one study reported that patients with alcohol dependence performed worse than healthy controls after outcome devaluation. Compared to controls, these patients displayed increased functional activation of the dorsal striatum (posterior putamen) during habit learning and less activation of the ventromedial prefrontal cortex and anterior putamen during goal‐directed learning [79]. However, another study combining outcome devaluation with drug‐associated conditioned stimuli observed no significant difference in goal‐directed behaviour between patients with AUD and healthy controls [67]. The inconsistent results may be due to specific details in laboratory tasks, including the presence of Pavlovian conditioned stimuli.

Another approach to assess goal‐directed versus habitual decision‐making examines the computations underlying model‐based versus model‐free behaviour. Model‐based decision making considers all short‐ and long‐term consequences of choices and can be assessed by the degree of forward planning in sequential decision‐making tasks [80]. A significant correlation between goal‐directed and model‐based behaviour was observed in healthy controls [81]. Model‐free decisions, on the other hand, do not take all possible choices into account but instead rely on observed experiences [1]. In the so‐called two‐step task, model‐based decision‐making means that subjects use a ‘model of the world’ and repeat actions that are commonly reinforced, even when they were punished in the last trial due to a rare transition. Conversely, model‐free behaviour is driven only by recent experiences of reward or punishment, which may be due to a ‘habitual stamping‐in of stimulus‐response associations, hypothesized to arise computationally from “model‐free” learning’ [82].

Dopamine plays a significant role in balancing model‐based and model‐free behaviour: In patients with Parkinson's disease, lack of dopaminergic medication impaired model‐based decision making [82]. In healthy individuals, higher dopamine synthesis in the ventral striatum was associated with more model‐based behaviour, greater coding of model‐based signatures in the lateral prefrontal cortex, and diminished coding of model‐free prediction errors in the ventral striatum [16]. Therefore, reduced dopamine synthesis capacity and D2 receptor availability, as observed among persons with AUD [63, 64, 65, 68, 69, 71, 72, 74, 75], may promote model‐free behaviour at the expense of model‐based, goal‐directed decision making. However, no significant impairment of model‐based decision making was found among individuals with AUD [70, 83]. On the other hand, lower model‐based behaviour, weaker hippocampal and stronger ventral striatal learning signals were associated with a decreased ability to intentionally reduce alcohol intake in real life among persons with AUD [84], pointing to gradual rather than effects of model‐based versus model‐free decision making.

Paradigms to assess model‐based versus model‐free behaviour may not best be suited to explore goal‐directed versus habitual decision making [85], because both model‐based and model‐free behaviour are rewarded and may hence be chosen rather than automatically activated. Another way to assess a tendency towards habitual and inflexible behaviour in persons with drug dependence is to measure performance when reward contingencies change, and the person has to reverse previously established choices. Indeed, it was observed that learning from reward and punishment feedback was impaired when assessing individuals with substance use disorders in reversal learning tasks, in spite of unimpaired working memory and attention [16, 86, 87]. Reversal learning parameters were not associated with alcohol intake in adolescents at ages 14, 16 and 18, suggesting that impairments observed in persons with AUD develop during chronic alcohol intake [88].

In nonhuman primate studies of reinforcement learning, firing of dopamine midbrain neurons reflects errors in reward prediction [89, 90]. In human studies, computational models can assess the respective size of this prediction error during reward learning and its association with functional activation in target areas of dopamine midbrain neurons, including the ventral striatum, where neural network activation is supposed to reflect phasic changes in dopamine firing [15, 91]. Using such reversal learning tasks, several studies with different modelling strategies failed to observe significant alterations in ventral striatal functional activation associated with reward prediction errors among patients with AUD [16, 87, 92, 93]. However, there were significant differences between patients with AUD and healthy controls regarding encoding of reward prediction errors in the medial prefrontal [92] and dorsolateral prefrontal cortex [93] and in functional connectivity between the striatum and dorsolateral prefrontal cortex [87].

To confirm that alterations in dopamine neurotransmission are associated with changes in functional activation elicited by reward prediction errors or alcohol cues, pharmacological challenge or multimodal imaging studies can be used [77, 94, 95]. Although effects of receptor blockade may be due to interactions between different neurotransmitter systems [96], multimodal imaging combining positron emission tomography with functional magnetic resonance imaging can directly assess correlations between dopamine function and neural activation [97]. However, such studies are rare in AUD. One such study observed that healthy controls displayed a significant negative correlation between dopamine synthesis capacity and reward prediction encoding in the ventral striatum, which was absent in patients with alcohol dependence [16]. In case of replication, this observation suggests that tonic (or at least long‐term) dopamine modulation of phasic reward prediction error encoding is impaired by excessive long‐term alcohol consumption.

Altogether, reversal learning studies indicate that behavioural flexibility is impaired in patients with AUD, who met the learning criterion of correct choices less often [92, 93] and achieved a lower number of reversal stages [16, 87]. Accordingly, these persons are able to learn new behavioural strategies but may need more time or support when having to reverse an established behaviour pattern.

It has been criticized that the habit theory of addictive behaviour implies that addicted persons are ‘intellectually impaired’ [98]. Indeed, theories that reify goal‐directed and habitual behaviour into separate and competitive systems and suggest that goal‐directed decision making is lost in persons with drug addiction could be used to stigmatize them or even question their right to self‐determination. However, we strongly feel that habit formation is not a pathological process but instead reflects a gradual change from ventral‐to‐dorsal striatal control that occurs whenever a behaviour becomes habitual [99], for example, when learning to drive a car. A recent meta‐analysis also supports the hypothesis of a gradual rather than categorical distinction between more goal‐directed versus habitual decision making [100]. Therefore, persons with drug dependence may show habitual drug consumption in some situations even if the general ability for model‐based decision making is not impaired.

## 4 Learning Mechanism Contributing to Relapse After Detoxification

Alcohol is a ‘dirty drug’ that induces neuroadaptations in a wide range of neurotransmitter systems, including GABAergic and glutamatergic neurotransmission [101, 102, 103]. When alcohol intake is suddenly stopped, recovery of neurotransmitter systems takes some time, and aversive withdrawal symptoms can promote relapse [9]. Aversive withdrawal reactions can also manifest as a conditioned reaction, a phenomenon mostly known from opiate withdrawal but also described in human AUD [104, 105]. Drug consumption during relapse can alleviate such aversive withdrawal states, thus negatively reinforcing alcohol intake.

The stimulating effects of alcohol intake on dopamine release cease immediately with abstinence, whereas neuroadaptations to chronic alcohol intake recover more slowly [106, 107]. During withdrawal, dopamine D2 receptors, synthesis and release were reduced and recovered during early abstinence [62, 68, 69, 108]. Also, a lower dopamine synthesis capacity (measured over 1 h) and reduced dopamine release (elicited by the application of a psychostimulant) were found in recently detoxified patients with alcohol dependence [19, 20, 22]. During late abstinence, dopamine D2 receptors appear to recover or even exceed normal levels [69, 109], whereas delayed recovery of dopamine D2 receptor sensitivity was associated with a poor treatment outcome [3, 24, 27, 72, 73, 110, 111]. A lower availability of dopamine D2 receptors in the ventral striatum was associated with increased activation of the medial prefrontal cortex [71] elicited by alcohol cues, which correlated with subsequent relapse [54, 112].

But why should a blunted dopamine transmission contribute to drug craving and the relapse risk during early abstinence? This may be due to the fact that a downregulated dopamine system will respond less strongly to natural reinforcers compared to drugs of abuse, which release more dopamine without rapid habituation [3, 113, 114]. There is indeed meta‐analytic evidence for reduced functional activation in the ventral striatum in response to the anticipation of nondrug reward [115]. Moreover, increased craving for alcohol was correlated with a reduced dopamine synthesis capacity and a lower availability of dopamine D2 receptors in the ventral striatum [108] and thalamus [116], as well as D3 receptors in the substantia nigra [117] among persons with AUD. Persons with a downregulated dopamine system may thus respond less strongly to natural reinforcers and crave drugs of abuse, which have an unusually strong effect on dopamine release [118] that can still reinforce further drug intake in spite of an overall blunted reward system.

With respect to Pavlovian mechanisms, extinction processes may be impaired, and exposure to alcohol cues can motivate persons with AUD to actively seek and consume alcohol in spite of conscious decisions to remain abstinent [7, 71]. Indeed, increased functional activation elicited by alcohol cues was associated with an increased relapse risk in several studies [54, 112, 119] and can be reduced by blockade of mu opiate receptors and modification of GABAergic neurotransmission [120, 121, 122, 123, 124, 125].

Pavlovian mechanisms can bias independent instrumental behaviour towards drug seeking. For example, one of our patients stated, ‘In the evening, when the sky turns gray, and I feel a little lonely, and I pass by these bars with the warm yellow light in the window and hear the clinking of glasses, I am lost’. A Pavlovian conditioned stimulus, for example, a cue that was regularly associated with drug reward (here, the clinking of glasses in a social ritual), can thus impact goal‐directed behaviour, including drug seeking and intake. Such effects of drug‐associated cues on instrumental behaviour can be explored using the so‐called Pavlovian‐to‐instrumental transfer (PIT) paradigm. In such PIT paradigms, Pavlovian conditioned cues or drug‐related stimuli are presented, and their impact on an unrelated goal‐directed behaviour is assessed. An increased effect of Pavlovian conditioned cues on instrumental behaviour was associated with an alcohol approach bias and increased activation of the ventral striatum [126].

However, several studies have failed to observe that drug‐related cues increase (appropriate or inappropriate) approach behaviour [127, 128]. Instead, it was observed that detoxified patients, who were able to abstain from alcohol intake for several months, stopped instrumental approach behaviour in the presence of alcohol cues, which was not the case among subsequent relapsers and healthy controls [129]. Humans reflect on their behaviour and verbal comments of our probands and patients during testing show that they pay attention to the potential meanings of Pavlovian cues presented in the background during goal‐directed behaviour. Those who used alcohol cues as warning signs fared better in the next 3 months compared to those who behaved just like control subjects without AUD [128]. A limitation of this observation is that the paradigm applied here was not able to distinguish between general and specific PIT effects, that is, between generally motivating effects of Pavlovian stimuli independent of the specific reward that can be obtained and Pavlovian stimuli associated with a specific reward that enhance a goal‐directed response towards the same reward [130]. Drug‐related cues may thus generally inhibit instrumental approach behaviour among alcohol‐dependent persons with a good treatment outcome yet may still elicit drug craving and seeking in vulnerable individuals [129].

Some theories suggest that drug‐related behaviour is ‘compulsive’ because it persists in spite of punishment [10, 78]. Here, addictive behaviour is understood as an ‘apparently irrational perseveration in maladaptive acts’ in spite of (immediate) personally negative effects, which are put in parallel to obsessive‐compulsive disorder and are proposed to reflect the ‘manifestation of a dimensional trait termed compulsivity’ [61]. However, in humans, the rewarding effects of drug intake are immediate, and aversive consequences are usually delayed, whereas in many animal models of compulsive drug intake, drug‐related actions are immediately punished [131]. To provide more ecologically salient models, aversive consequences of drug intake can be delayed by several hours [132], be provided in a probabilistic manner or only anticipated by presentation of conditioned cues [133, 134, 135]. However, it is difficult to model the long delay of aversive social and physical consequences of harmful alcohol use [136, 137]. Significant differences in the neurobiological correlates of OCD and addiction [131, 138, 139] were detected, for example, increases versus decreases in frontal glucose utilization or differences in functional connectivity [131, 138, 139]. Furthermore, subjective experiences, including the ability to resist obsessions and compulsions on the one hand and drug craving and seeking on the other, differ clinically [140], which questions the hypothesis that addictive behaviour in humans should be labelled ‘compulsive’.

Independent of whether addictive behaviour is understood as a manifestation of a behavioural compulsivity trait, meta‐analytic evidence points to an impairment of cognitive functions during detoxification and the first year of abstinence [141]. Neurotoxic effects of chronic alcohol intake can cause brain atrophy and impair cognition [142, 143], which recover during long‐term abstinence [141, 142]. Several studies did not find a significant impairment in goal‐directed decision‐making [70, 83] or working memory performance (as an example of an executive dysfunction) [144] and a meta‐analysis failed to demonstrate a significant difference in behavioural inhibition among persons with substance use disorders [145]. Therefore, although there is evidence for neurotoxic effects of long‐term alcohol intake on cognitive performance, recovery and relevance for the maintenance of drug intake remain to be explored in more detail.

Alternatively, it was suggested that drug use is an individual (bad) choice not explained by effects of drug cues and habit formation but instead associated with negative affect and limited alternative choices, for example, in socially deprived situations [98, 146]. Although poverty, social isolation and discrimination are indeed important risk factors for excessive drug intake [21], attributing persistent drug intake exclusively to goal‐directed behaviour [147] runs the risk of ignoring unintended behavioural biases towards drug taking. Subjects with drug addiction are confronted with the particularly difficult task of changing drug‐taking habits associated with drug‐induced alterations in reinforcement learning [114]. As the speed of recovery of dopamine neurotransmission was associated with poor treatment outcome [72, 73], persons with more severe alterations of this system appear to have more difficulties remaining abstinent and require support to acquire alternative behavioural strategies.

Limitations of the assessment of learning mechanisms in AUD include different paradigms and modelling strategies and rather small effect sizes. More complex paradigms with various methods of outcome devaluation [61, 62] or high cognitive demands [148] appear to provide less reliable results than comparatively straightforward tasks assessing reversal learning [16, 86, 87] or an alcohol approach bias [59, 60]. Moreover, explained variances and effect sizes tend to be high in discovery samples but lower in replications. For example, functional activation elicited by alcohol cues in the anterior cingulate and medial prefrontal cortex predicted more than 80% of the subsequent relapse risk, with no significant contribution of craving severity or duration of abstinence [112]. However, such findings are discovered exactly because they are highly significant yet tend to be modulated by a series of factors, including drug availability, comorbid tobacco dependence and visual confounds [149, 150, 151, 152]. Moreover, alterations in dopamine function vary with brain regions and methodology; for example, peak differences between detoxified patients with AUD and controls were found in the left medial caudate, with an effect size of *d* = 0,76, whereas differences in other brain regions did not reach significance [153]. Standardization of paradigms and imaging techniques could help to increase replicability.

In summary, persons with AUD deserve intensive therapeutic efforts that take their individual capacities into account [154, 155, 156]. It might be helpful to individually adjust the frequency or amount of positive feedback to personal differences in learning mechanisms, as indicated, for example, by learning mechanisms in reversal paradigms [48, 72, 73]. Digital tools that assess cognitive capacity in real life, also in challenging and stressful situations, may help to individually adapt behavioural interventions and social support [157, 158, 159].

## 5 Social and Therapeutic Consequences of Studying Learning Mechanisms in AUDs

Can a focus on learning mechanisms help reduce the stigma associated with AUD? Persons with drug use face considerable stigmatization [160]. Drug use is traditionally attributed to impulsive or otherwise poor decision‐making [161], which is often understood as a stable trait characterizing persons with AUD [162]. Stigma is promoted by so‐called ‘essentialist’ beliefs in clear‐cut categorical differences between persons with and without addiction, even if such putative distinctions are attributed to ‘basic identifiable traits’ or putatively addictive ‘personality types’ rather than genetic differences [163]. However, impulsivity is a multifaceted construct whose different aspects (including discounting of a delayed reward or inhibition of ongoing predominant behaviour) are often only poorly correlated [164]. Moreover, potentially heritable personality traits interact with experiences of early trauma, poverty, social isolation and discrimination [21, 164, 165]. Explaining how learning can be impaired in persons with AUD can help to explain why it is difficult to change addictive behaviour and thus counter the idea that individuals with substance use disorders are personally responsible for continuous drug consumption despite aversive consequences [166]. However, a meta‐analysis did not confirm the hypothesis that neurobiological explanations reduce the blame attached to persons with mental disorders [167]. Instead, neurobiological explanations of mental disorders often tend to promote social distance, potentially because they activate essentialist intuitions associated with prejudices [167]. A focus on learning mechanisms, therefore, needs to clearly state how (unintended) drug effects on the central nervous system [168] gradually bias behaviour towards drug intake and can render it particularly difficult for persons with drug dependence to change their behaviour. However, this is only one possible interpretation of neurobiological findings, and a fair and humane treatment of persons with drug addiction must be fought for again and again by users, relatives and professionals. Successful interventions against stigma need to promote identification with persons with mental illness [169] and should thus include persons with a history of drug consumption as experts by experience. With respect to therapeutic consequences, most therapeutic interventions use some form of cognitive behavioural therapy (CBT) or related approaches [154, 155, 156, 170], which rely on learning mechanisms to understand how behaviour was acquired and can be changed [171, 172]. Such behavioural interventions may profit from adjustment to individual learning mechanisms, which can be quantified, for example, in reversal learning paradigms [16, 86, 87]. Beyond CBT, adjunctive occupational therapy can be effective [173], also with respect to affective comorbidities [174]. Natural settings provide real‐life triggers that can challenge clients to gain control in diverse settings. Participants of recovery programmes from substance use, which include occupational therapy, reported that they need additional experiences outside the rehabilitation centre to practise the skills learned during group intervention [175]. Accordingly, initial findings suggest that persons who attended occupation‐based programmes experience fewer episodes of relapse [176] and tend to disengage from activities associated with previous substance use [177].

Regarding pharmacological interventions, both tonic stimulation and inhibition of dopamine D2 receptors failed to improve the relapse risk among persons with alcohol dependence [4, 5]. However, it was recently reported that a glucagon‐like peptide‐1 receptor agonist (GLP1RA) can restore impaired associative learning among persons with obesity [178], hypothetically via modulation of phasic dopamine release in the Nucleus accumbens/ventral striatum [179]. In a randomized clinical trial, the application of a GLP‐1RA significantly reduced craving and drinks per drinking day [180]. The GLP‐1RA dulaglutide was associated with reduced alcohol intake in another randomized controlled trial, whereas no significant effect was observed following exenatide medication [181]. A recent meta‐analysis observed a lower rate of AUD among persons treated with GLP‐1RAs [182]. Future studies should assess GLP‐1RA effects on cue response and reinforcement learning among persons with AUD. In prospective trials, most people with AUD relapse within the first weeks of abstinence [54, 72]. As brain atrophy, dopamine dysfunction and cognitive impairments recover during longer term abstinence [69, 72, 109, 141, 142], it is important to offer immediate care following detoxification. However, persons with AUD face substantial barriers to treatment, and following detoxification, therapy for addictive behaviour can be substantially delayed [183]. Reviewing learning mechanisms in AUD suggests that impairments in reward learning recover during abstinence and may already improve during reduced intake [136], whereas social isolation, exclusion and discrimination can impair dopamine neurotransmission and increase the risk of developing and maintaining AUD [21]. Beyond individual risk factors, poverty in the neighbourhood contributes to distress [184] and was associated with increased levels of pollution [185], which in turn can promote somatic comorbidity and poor treatment outcomes [186]. Therefore, treatment barriers need to be reduced, and community mental health care should be promoted. Individuals with drug use problems deserve equal access to the healthcare system, like all other persons with mental health issues, and social interventions aimed at reducing drug use need to include a focus on modifiable factors in the lived environment. This requires a participatory approach that includes individuals with lived experiences when designing research and interventions and fighting stigma and social exclusion.

## Author Contributions

Andreas Heinz conceptualized the study and supervised the project. Selin Icoez and Yvonne Treusch contributed to writing and editing of the manuscript. Gaetano Di Chiara contributed to writing, review, and editing, including critical revisions of the manuscript. Melissa G. Halil wrote the original draft and contributed to writing, review, editing, and project administration. All authors reviewed and approved the final manuscript.

## Funding

The authors have nothing to report.

## Conflicts of Interest

The authors declare no conflicts of interest.

## Data Availability

Data sharing is not applicable to this article, as no datasets were generated or analysed during the current study.
